# In ovo feeding of l-arginine and selenium nanoparticles influences post-hatch growth, muscle development, antioxidant status, and meat quality in slow-growing chickens

**DOI:** 10.1093/jas/skae290

**Published:** 2024-09-24

**Authors:** Pramin Kaewsatuan, Thanidtha Morawong, Panpan Lu, Anyanee Kamkaew, Amonrat Molee, Wittawat Molee

**Affiliations:** School of Animal Technology and Innovation, Institute of Agricultural Technology, Suranaree University of Technology, Nakhon Ratchasima 30000, Thailand; School of Animal Technology and Innovation, Institute of Agricultural Technology, Suranaree University of Technology, Nakhon Ratchasima 30000, Thailand; School of Animal Technology and Innovation, Institute of Agricultural Technology, Suranaree University of Technology, Nakhon Ratchasima 30000, Thailand; School of Chemistry, Institute of Science, Suranaree University of Technology, Nakhon Ratchasima 30000, Thailand; School of Animal Technology and Innovation, Institute of Agricultural Technology, Suranaree University of Technology, Nakhon Ratchasima 30000, Thailand; School of Animal Technology and Innovation, Institute of Agricultural Technology, Suranaree University of Technology, Nakhon Ratchasima 30000, Thailand

**Keywords:** meat quality, muscle growth, in ovo feeding, l, -arginine, selenium nanoparticle, slow-growing chicken

## Abstract

This study investigated the effects of in ovo feeding (**IOF**) of l-arginine (**L-Arg**), selenium nanoparticles (**SeNP**), and a combination of L-Arg and SeNP on the hatchability, post-hatch growth, muscle development, antioxidant status, and meat quality of slow-growing chickens. On day 18 of incubation, a total of 960 fertilized eggs with similar weights were randomly assigned to 4 treatment groups with 4 replicates of 60 eggs each: (1) non-injected control group (Control), (2) injected with 1% of L-Arg (**IOF_L-Arg**), (3) injected with 0.3 µg/egg of SeNP (**IOF_SeNP**), and (4), injected with 1% of L-Arg and 0.3 µg/egg of SeNP (**IOF_L-Arg + SeNP**). A completely randomized design was used. After hatching, 640 mixed-sex chicks were allocated to 4 treatment groups and split into 4 replicate pens (40 birds per pen). All groups of chicks were fed with commercial feed ad libitum until they reached 63 d of age and were subsequently weighed and slaughtered. The results of the present study showed that hatchability was similar among treatments. Final BW or breast muscle yield was not affected (*P* > 0.05) by IOF treatment. Chickens treated with IOF_L-Arg + SeNP exhibited decreased feed conversion ratio, drip loss, and increased protein content in breast meat (*P* < 0.05). The IOF_L-Arg + SeNP group exhibited a higher density of breast muscle fibers than the control group (*P* < 0.05). Overall, in ovo feeding of L-Arg combined with SeNP resulted in improved feed efficiency and enhanced antioxidant capacity at hatch without any adverse effects on chicken hatchability, health, or subsequent growth. Furthermore, meat from chickens in the IOF_L-Arg + SeNP group exhibited a preferable texture with a higher protein content.

## Teaser

Chickens receiving both L-arginine and selenium nanoparticles through in ovo feeding method exhibited improved feed conversion ratio and muscle fiber development without adverse effects on hatchability and growth performance. Additionally, the breast meat from chickens in this treatment also showed a preferable texture with higher protein content.

## Introduction

The meat of slow-growing chickens has gained global attention from consumers concerned about healthy meat and welfare. However, these birds have a lower breast meat yield than commercial broilers, limiting their production efficiency and market competitiveness (Katekhaye, 2019). Thus, new strategies and technologies aimed at optimizing muscle gain are crucial for slow-growing chicken production.

Recently, skeletal muscle development was enhanced prior to hatching by nutrient manipulation through in ovo feeding (**IOF**; Subramaniyan et al., 2020; Xu et al., 2021; Muyyarikkandy et al., 2023). IOF delivers critical micronutrients directly to the developing embryo (Givisiez et al., 2020). L-Arginine **(L-Arg)**, an essential amino acid, serves as a substrate for the biosynthesis of biologically important molecules such as proteins, ornithine, proline, polyamines, nitric oxide (**NO**), glutamate, glutamine, and creatine (Khajali and Wideman, 2010). Numerous research demonstrated that IOF of L-Arg can improve protein deposition and subsequent breast muscle growth (Yu et al., 2018), stimulate myoblast differentiation (Li et al., 2016), increase intestinal weight, and promote the morphological development of the small intestine of chicks by activating the mTOR pathway (Gao et al., 2018). Furthermore, our previous study demonstrated that IOF of L-Arg positively influences the antioxidant status and myogenic genes of slow-growing chickens post-hatch (Lu et al., 2022a, 2022b).

Antioxidant components, such as selenium (**Se**), are also primarily administered to fertilized eggs through in ovo method to enhance antioxidant defenses during embryogenesis, as these components effectively reduce free radical oxygen (Huang et al., 2012). Nowadays, much attention has shifted to the use of selenium nanoparticles (**SeNP**) as animal feed additives because of their new features, for example, high surface activity, strong solubility and mobility, high bioavailability, and low toxicity (Hu et al., 2012). Previous studies have indicated that the nanoform of Se is more effective than the inorganic and organic forms (Zhang et al., 2008). The nano-Se used in conventional or in ovo methods has been proven to promote growth rate, immune response, and overall performance of chickens (Hassan, 2018; Bień et al., 2023). Given the beneficial and effective effects of L-Arg and SeNP, it can be assumed that in ovo feeding of L-Arg combined with SeNP is a more efficient way to modulate post-hatch overall performance and muscle growth.

This study also focused on the Korat chickens (**KRC**), an important alternative meat-type chicken breed. In Thailand, KRC meat is prized for its nutritional value and unique texture, making it an important local poultry protein source (Kaewsatuan et al., 2022); however, knowledge of these chickens is still rare. Thus, the present study aimed to determine the effectiveness of using L-Arg, SeNP, or a combination of L-Arg and SeNP as an in ovo feeding method in KRC slow-growing chickens in terms of hatchability, post-hatching performance, muscle growth and characteristics, antioxidant capacity, and meat quality to provide a basis for developing early nutritional strategies in poultry.

## Materials and Methods

### Ethics statement

All experimental procedures were approved by the Ethics Committee on Animal Use of Suranaree University of Technology (**SUT**), Nakhon Ratchasima, Thailand (Approval ID: 18/2560; U1-02633-2559).

### Preparing L-Arg and SeNP solutions

L-Arg (1%) was prepared as previously described (Yu et al., 2018). The Arg solution concentration of 1% was chosen based on prior research conducted by Gao et al. (2017). Briefly, 10 g of L-Arg (#A5006; Sigma-Aldrich Inc., St. Louis, MO, USA) was dissolved in 100 mL of 0.75 NaCl diluent solution to obtain a 1% (w/v) solution.

**Figure 1.**
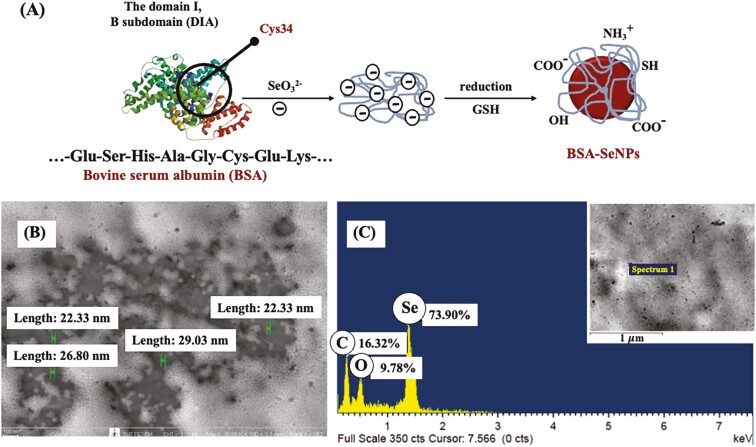
The binding of BSA and SeNPs at the domain I, B subdomain in the area of Cys34 (A), scanning electron microscopy (SEM) image of the selenium nanoparticles produced by BSA (5,000 × magnification) (B), and the energy dispersive spectroscopy (EDS) spectra showed the purity of the Se substance (C).

Based on the nutrient requirements for broilers reported by Leeson and Summers (2005), we selected a selenium injection concentration of 0.3 µg/mL/egg. Bovine serum albumin (**BSA**; Sigma-Aldrich)-coated selenium nanoparticles (**SeNP**) were prepared as previously described (Zhang et al., 2001). Sodium selenite (5 mL of 25 mM) was mixed with 20 mL of 25 mM reduced glutathione (**GSH**) containing 200 mg BSA. The pH of the mixture was adjusted to 7.2 with 1.0 M sodium hydroxide, immediately forming nano-red elemental Se and oxidized GSH. The final solution containing SeNP was lyophilized and stored at room temperature. Figure 1A presents an overview of the SeNP synthesis. Scanning electron microscopy revealed that the SeNP size was between 23.27 and 27.77 nm (JEOL JSM 7800F, Carl Zeiss Auriga, Germany; Figure 1B). Energy dispersive spectroscopy (Oxford Instruments Ltd, Abingdon, Oxfordshire, UK) confirmed the SeNP purity (Figure 1C), and the chemical composition of the sample yielded approximately 73.90% selenium, 16.33% carbon dioxide, and 9.78% oxygen.

### IOF procedure and incubation

The experimental eggs belonged to the slow-growing KRC breed, a crossbreed between Leung Hang Khao (**LHK**) males and SUT synthetic line females, and were collected from the SUT farm, Nakhon Ratchasima, Thailand. All eggs were supplied from a 35-wk-old LHK and a 28-wk-old SUT breeder flocks with an average weight of 48.75 ± 0.49 g. The eggs were randomly placed in a hatching incubator (Model: Petersime NV, Centrumstraat 125-9870 Zulte (Olsene), Belgium) at 37.67 ± 0.20 °C and 62% relative humidity. The IOF procedure was performed on day 18 of incubation. All eggs were candled to select embryonated eggs and eliminate unfertilized or nonviable eggs. Subsequently, 960 embryonated eggs of similar weight were assigned to 4 treatment groups with 4 replicates and 60 eggs per replicate each based on a completely randomized design. The treatment groups were (1) a non-injected control group, (2) injected with 1% L-Arg solution (IOF_L-Arg), (3) injected with 0.3 µg SeNP/egg solution (IOF_SeNP), and (4) injected with 1% L-Arg (0.05 g/egg) + 0.3 µg SeNP/egg solutions (IOF_L-Arg + SeNP). The treatment solution was freshly prepared on the day of injection. A hole was created at the large end surface of the eggs, and 0.5 mL of the solutions were injected into the amnion using a 22-gauge needle. Immediately, after the IOF procedure, each well was sealed with liquid paraffin, and egg incubation was continued until hatching.

### Housing the birds

After hatching, 1-d-old mixed-sex chicks from each of the 4 treatment groups were pooled and weighed. In total, 640 mixed-sex chicks with similar weights close to the average BW of their pooled group were distributed in 16th-floor pens for 4 treatments in open-sided housing, and each pen was regarded as a replicate (40 chicks per pen; 160 chicks per treatment group). Commercial feed (Charoen Pokphand Co., Ltd., Nakhon Ratchasima, Thailand), and freshwater were provided ad libitum until the chicks reached 63 d of age. The chicks were maintained in an open indoor housing with an automatic fan for ventilation where the temperature ranged from 30 to 37 °C, and the photoperiod was 12 h throughout the rearing period. Vaccinations were administered as follows: a Marek’s disease vaccine on the first day, followed by Newcastle disease vaccine and infectious bronchitis vaccinations on the 7th and 22nd days. Additionally, a Gamboro vaccine was given on the 14th day. Table 1 provides the details of the basal diet of the selected chickens, provided by AOAC (2006).

**Table 1.** Analyzed nutrients of the basal diet in each feeding phase

Analyzed compositions (%)	0 to 21 d	22 to 42 d	43 to 63 d
Dry matter	93.84	93.51	94.21
Gross energy, GE (kcal/kg)	3,946.28	4,081.03	4,111.77
Crude protein	22.72	20.46	18.65
Crude fat	5.20	6.74	6.66
Crude fiber	3.44	3.45	3.55
Ash	4.70	4.58	4.19
l-Lysine	1.78	1.43	0.92
l-Arginine	1.58	1.13	0.55
dl-Methionine	0.34	0.25	0.28
l-Threonine	1.01	0.85	0.73
l-Valine	1.32	1.05	0.72
Selenium (mg/kg)	0.60	0.33	0.18

### Hatching parameters

On the day of hatching, the weights of all hatched chicks were recorded (60 eggs/replicate), and the hatchability percentage was calculated for all treatment groups using the following equation:


$$\begin{array}{l} Hatchability\;(\%)=\frac{Number\;of\;hatched\;eggs\;on\;21st\;day}{Number\;of\;fertiled\;eggs\;that\;were\;in\;IOF}\times \;100 \end{array}$$

### Growth performance

BW and feed intake (**FI**) on day 63 were measured to calculate body weight gain (**BWG**) and feed conversion ratio (**FCR**). Dead birds were recorded daily to determine the mortality rate.

### Sample collection

On days 1 (D0), 7 (D7), 14 (D14), 21 (D21), 42 (42), and 63 (D63) post-hatching, 2 males and 2 females from each replicate (16 chickens per treatment) were randomly sampled after 12 h of fasting. The chickens were weighed and sacrificed by electric stunning, followed by exsanguination, de-feathering, and evisceration. Immediately after dissection, portions of the breast muscles were weighed and collected for morphological analysis. Additionally, breast muscle samples from birds euthanized on D0 were stored at −80 °C for enzyme activity analysis.

On the final slaughter day 63, 3 mL blood samples were collected from the chickens via the jugular vein. The blood was stored in non-heparinized tubes at −20 °C for subsequent biochemical analysis. After blood collection, the breast, kidney, and liver were weighed and stored at −20 °C until further analysis. Furthermore, the remaining breast muscle samples were stored at 4 °C for meat quality assessment and at −80 °C for enzyme activity analysis.

### Serum biochemistry

After clotting, the blood samples collected on the slaughter day were centrifuged at 3,000 × *g* for 30 min to obtain the serum. The serum samples were subsequently transferred to microtubes, stored in a freezer for 24 h, and analyzed at SUT Hospital, Nakhon Ratchasima, Thailand. The total serum protein concentration was determined using the biuret method. Serum albumin levels were measured using the bromocresol green method. Serum globulin levels were calculated as the difference between the total protein, albumin, and uric acid levels using the Trinder enzymatic method.

### Tissue selenium depositions

Tissue selenium depositions were measured on D63. Briefly, 0.5 g of breast, liver, and kidney were digested in a mixture of 1 mL hydrogen peroxide (**H**_**2**_**O**_**2**_; 30% pro analysis, Merck, Darmstadt, Germany) and 4 mL nitric acid (**HNO**_**3**_; 65% Suprapur, Merck, Darmstadt, Germany) using a microwave (Automated Microwave Digestion System Discover SP-D 80, Jinan Hanon Instruments Co., Ltd., China). After sample digestion, the digestion solution was diluted to 25 mL with ultrapure water and the selenium content was measured using an inductively coupled plasma source mass spectrometer (7500CE ICP-MS, Agilent Technologies, Inc., USA). The chemical and biochemistry analysis laboratory of the SUT, Nakhon Ratchasima, Thailand provided the selenium stock standard solutions (1,000 µL/mL in 4% HNO_3_).

### Measuring enzyme activity

Enzyme activity was determined between D0 and D63. Breast muscle samples (0.3 g) were homogenized with ice-cold 0.9% sodium chloride buffer using a T25 digital Ultra-Turrax homogenizer (IKA Co., China) and subsequently centrifuged at 4,550 × *g* for 15 min at 4 °C. GSH-Px activity was measured by an enzymatic assay of glutathione peroxidase (EC 1.11.1.9, Sigma). The absorbance readings were monitored using a microplate spectrophotometer (Thermo Fisher Scientific, Finland) at 340 nm.

### Analyzing meat quality

Breast meat quality was assessed on D63 using the following parameters: ultimate pH, color, drip loss, cooking loss, shear force, and proximate composition. The ultimate pH of the breast muscle was determined 24 h after slaughter using a pH meter (HI-99163; Hanna Instruments, Inc., UK). Meat lightness (L*) was measured using a colorimeter (Chroma Meter CR-300; Minolta, Japan) on the dorsal surface of the left breast muscle 24 h postmortem. The surface was exposed to air at room temperature (25 °C) for 30 min before determining meat color. Each value was the average of 3 separate points for each sample. For drip loss, the samples were cut into pieces (1.5 × 3.0 × 0.5 cm^3^) weighing approximately 7 g, which were weighted, placed in a plastic bag, stored in a chilling room at 4 °C for 24 h, and then reweighed. Percentage drip loss was calculated using the following equation:


$$\begin{array}{l} Drip\;loss\;(\%)=\left(\frac{weight\;before\;storage-weight\;after\;storage}{weight\;before\;storage}\right)\;\times \;100 \end{array}$$

Cooking loss was measured by weighing a 1.5 × 3.0 × 0.5 cm^3^ cut of breast meat, weighing about 25 g, placing it in a vacuum-sealed plastic bag, and cooking for 3 h in a water bath at 80 °C until the targeted core temperature reached 71 °C. The samples were subsequently cooled to room temperature, dried, and reweighed to calculate the cooking loss percentage using the following formula:


$$\begin{array}{l} Cooking\;loss\;\;\left(\%\right)=\left(\frac{weight\;before\;cooking-weight\;after\;cooking}{weight\;before\;cooking}\right)\;\times \;100 \end{array}$$

Cooked samples were used for shear force analysis. The samples were cut into pieces (1.0 × 2.0 × 0.5 cm^3^), with fibers parallel to the long axis and sheared using TA-XTplus Texturometer (Stable Microsystem Ltd., Surrey, UK). The test speed was set to 2 mm/s. Each value represents an average of at least three measurements. Finally, proximate composition analysis was conducted on the same 64 breast muscle samples to evaluate moisture, crude protein, total fat, and ash content in triplicates (AOAC, 2006).

The total collagen content was determined using the hydroxyproline assay. A 50-mg sample was hydrolyzed with 7 M NaOH at 121 °C for 40 min. The hydrolysate was neutralized with 3.5 M sulfuric acid (**H**_**2**_**SO**_**4**_), ﬁltered through Whatman number 4 filter paper, diluted with distilled water to a final volume of 200 mL, and reacted with chloramine T solution and Ehrlich’s reagent. The sample and hydroxyproline standard absorbance (Sigma–AldrichCo.) were measured at 560 nm using a spectrophotometer. The hydroxyproline contents in the standards and hydrolysate were calculated from the standard curve and subsequently converted to collagen content as follows:


$$\begin{array}{l} Collagen\;\left(\frac{mg}{g\;sample}\right)=hydroxyproline\;\left(\frac{mg}{g\;sample}\right)\;\times \;7.25 \end{array}$$

### Morphological analysis

Breast muscle was obtained by carefully dissecting approximately 1 × 2 × 0.5 cm of the muscle. Each sample was placed in 10% (vol/vol) buffered formalin fixative (05-011005Q; Bio-Optica, Italy) and stored at 25 °C for at least 24 h. After fixation, the sample was dehydrated through a graded series of alcohols and cleared by using a Tissue Processor (Model: ATP140 Amos scientific). The samples were then embedded in paraffin, cross-sectioned into 3-μm sections, and mounted on a microscope slide. Before staining with hematoxylin and eosin, the muscle tissue sections were incubated at 55 °C for 30 min and then rehydrated for 10 min in xylene mix RPE of isomers, 4 min in 100% ethanol (Dehyol assulute:06-10077E), 4 min in 95% ethanol, and 5 min in distilled water. After dehydration, the slides were placed in Mayer’s hematoxylin for 5 min. The sections were then rinsed in gentle running tap water for 5 min and transferred to eosina Y solution acquosa for 2 min. After staining, the slides were dehydrated back through the graded series of alcohols and Xylene mix RPE of isomers. The stained sections were analyzed for muscle morphology with a light microscope (Olympus CX21, USA) and ZEN software (Axis Cam ERc5s-Zen lite, 2012). Images of the slide were captured using an optical microscope at 40× magnification. Muscle fiber diameter were measured with ImageJ software (Schneider et al., 2012) in the representative regions within a 100 μm^2^ area.

### Statistical analysis

Descriptive statistics were applied, and all results are presented as mean ± standard error of the mean. Statistical analysis was performed by one-way ANOVA using SPSS (version 24.0; SPSS Inc., Chicago, IL, USA), followed by Tukey’s multiple tests. Statistical significance was set at *P* < 0.05.

## Results

### Embryonic growth

The hatchability of each IOF treatment was > 85%, and the differences between the treatments were statistically insignificant (*P* > 0.05). Additionally, the body weights of 1-d-old chicks did not differ between groups. The mean body weights were 44.52 g for the control group, 43.99 g for the IOF_L-Arg group, 43.89 g for the IOF_SeNP group, and 44.67 g for the IOF_L-Arg + SeNP group (SEM = 0.121; *P* > 0.05).

### Growth performance and feed efficiency

Table 2 summarizes the final BW, BWG, FI, and FCR values of the treatment groups. The differences in the final BW, BWG, or FI among the treatments were statistically insignificant (*P* > 0.05). However, the FCR was lower in the IOF_SeNP and IOF_L-Arg + SeNP groups (*P* < 0.05) than in the other groups.

**Table 2.** Effect of in ovo l-arginine and selenium nanoparticles on the growth performance of slow-growing Korat chickens at 63 d of age^1^

Parameters	In ovo feeding treatments^2^				SEM^3^	*P*-value
	Control	L-Arg	SeNP	L-Arg + SeNP		
Body weight gain, BWG (g)	1,232.60	1,149.57	1,274.56	1,236.31	43.170	0.074
Final body weight (g)	1,277.12	1,193.56	1,318.45	1,280.88	18.042	0.074
Feed intake, FI (g/d)	2,858.11	2,723.27	2,782.20	2,739.09	88.680	0.458
Feed conversion ratio, FCR	2.32^B^	2.37^B^	2.18^A^	2.22^A^	0.029	<0.01

^1^Each mean represents values from 4 replicates (40 birds/replicate).

^2^Control, non-injected control group; L-Arg, 1% l-arginine; SeNP, 0.3 µg selenium nanoparticles.

^3^Standard error of the mean.

### Breast muscle weight, muscle fiber density, and fiber diameter

**Figure 2.**
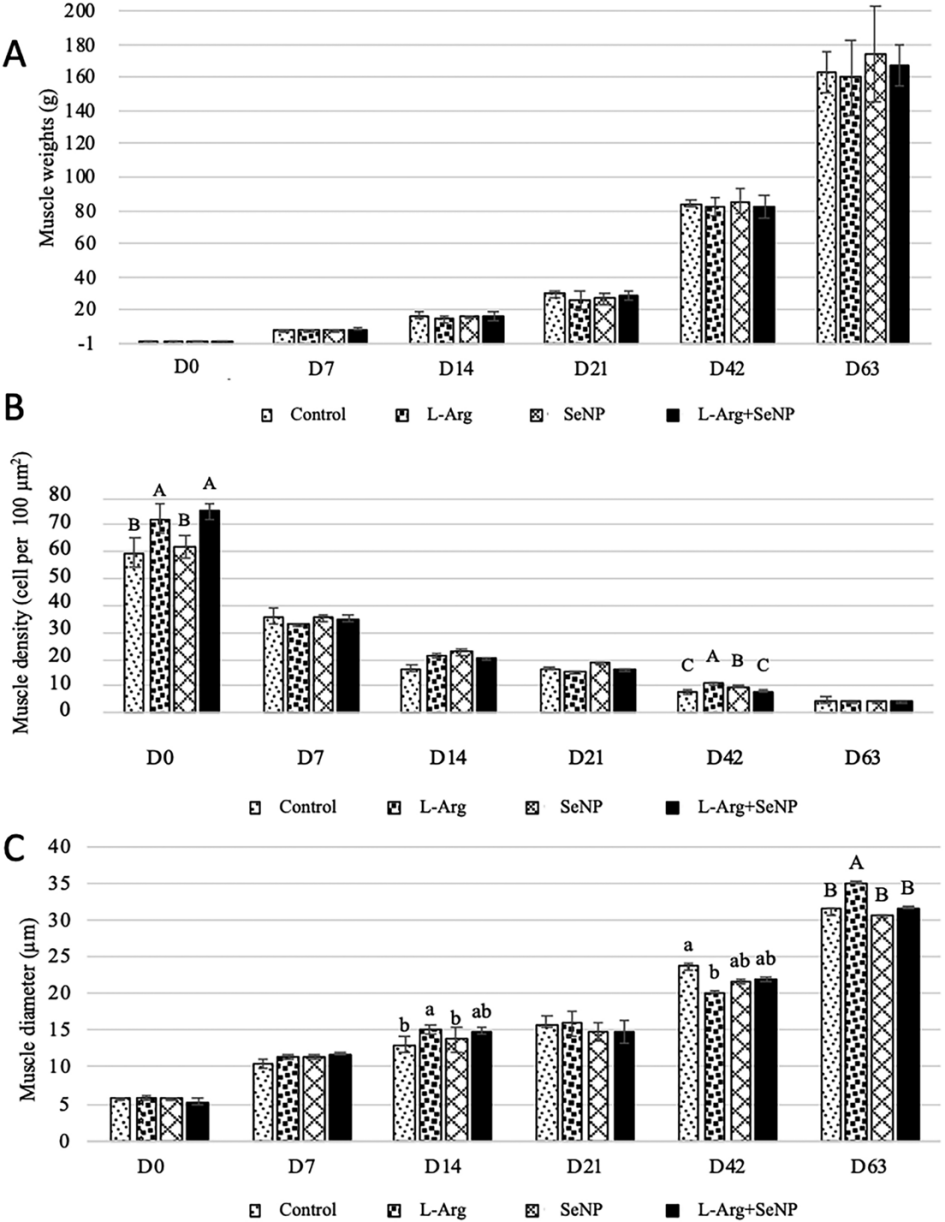
Effect of in ovo feeding of L-arginine and selenium nanoparticles on muscle development of Korat chicken. (A) Breast muscle weight, (B) breast muscle density, and (C) breast muscle diameter. A, B Means within column carrying no common superscripts are significantly different at *P* < 0.01. a, b Means within columns carrying no common superscripts are significantly different at *P* < 0.05. The data were presented as mean ± SEM (*n* = 16/treatment; 4 samples/replicate).

There was no significant effect of treatment on breast muscle weight (*P* > 0.05; Figure 2A). However, at hatching, the breast muscle fiber density (Figure 2B) was significantly increased in the IOF_L-Arg and IOF_L-Arg + SeNP groups compared with the control group (*P* < 0.01). While the IOF_SeNP group showed no significant difference from the control. On day 42, the breast muscle fiber density in the IOF_L-Arg group was the highest, whereas that in the control and IOF_L-Arg + SeNP groups was the lowest (*P* < 0.01).

Figure 2C illustrates the muscle fiber diameters of all treatment groups. On day 14, compared to the control group, the IOF_L-Arg group exhibited a significant increase in the muscle fiber diameter (*P* < 0.05), whereas the other IOF treatments did not differ from the control group. The largest diameters were observed in the breast muscle fibers of the control and IOF_L-Arg group on days 42 and 63 (*P* < 0.05, *P* < 0.01, respectively).

### Meat quality

Table 3 summarizes the breast meat quality and chemical composition across all treatments. The differences in most of the parameters, except for water, protein content, percentage of drip loss, and shear force were statistically insignificant. The percentages of water, protein content, and shear force in the breast muscle of the control group were significantly lower than those of the other IOF treatment groups (*P* < 0.05). The highest percentage of drip loss was observed in the breast muscles of the control group.

**Table 3.** Effect of in ovo feeding of l-arginine and selenium nanoparticles on the chemical compositions and meat quality of the breast meat of slow-growing Korat chickens (Pectoralis major)^1^

Parameters	In ovo feeding treatments^2^				SEM^3^	*P*-value
	Control	L-Arg	SeNP	L-Arg + SeNP		
Water content (%)	73.81^a^	71.94^ab^	71.55^b^	71.63^ab^	0.346	0.029
Protein content (%)	24.26^D^	25.72^C^	26.44^B^	27.29^A^	0.295	<0.01
Fat content (%)	1.57	1.36	1.41	1.36	0.067	0.938
Ash content (%)	6.12	5.58	6.46	5.12	0.186	0.194
Total collagen content (mg/g)	1.51	1.64	1.39	1.62	0.042	0.136
Lightness (L*)	51.70	51.09	52.31	51.89	0.545	0.906
Ultimate pH (pHu)	5.76	5.80	5.74	5.77	0.016	0.493
Drip loss (%)	12.42^A^	10.27^B^	10.60^AB^	9.52^B^	0.355	<0.01
Cooking loss (%)	24.47	25.16	23.76	23.36	0.317	0.231
Shear force (kg)	2.03^b^	2.38^a^	2.31^ab^	2.10^ab^	0.050	0.018

^1^Each mean represents values from 4 replicates (4 samples/replicate).

^2^Control, non-injected control group; L-Arg, 1% l-arginine; SeNP, 0.3 µg selenium nanoparticles.

^3^Standard error of the mean.

^A,B^Means within a row carrying different superscripts indicate significant differences at *P* < 0.01. ^a,b^Means within a row carrying different superscripts indicate significant differences at *P* < 0.05.

### Serum biochemical profiles

The effect of IOF treatment on the serum biochemical profiles. The effects for total protein, albumin (**A**), globulin (**G**), the A/G ratio, or uric acid were statistically insignificant in any of the treatment groups compared to the control (*P* > 0.05). Total protein levels ranged from 3.15 to 3.29 g/dL (SEM = 0.080; *P* = 0.597), and albumin levels ranged from 1.10 to 1.13 g/dL (SEM = 0.027; *P* = 0.977). Globulin levels were between 2.03 and 2.16 g/dL (SEM = 0.060; *P* = 0.827), while the A/G ratio varied from 0.48 to 0.52 (SEM = 0.014; *P* = 0.454). Uric acid levels ranged from 3.04 to 3.61 mg/dL (SEM = 0.218; *P* = 0.853).

### Selenium deposition

**Figure 3.**
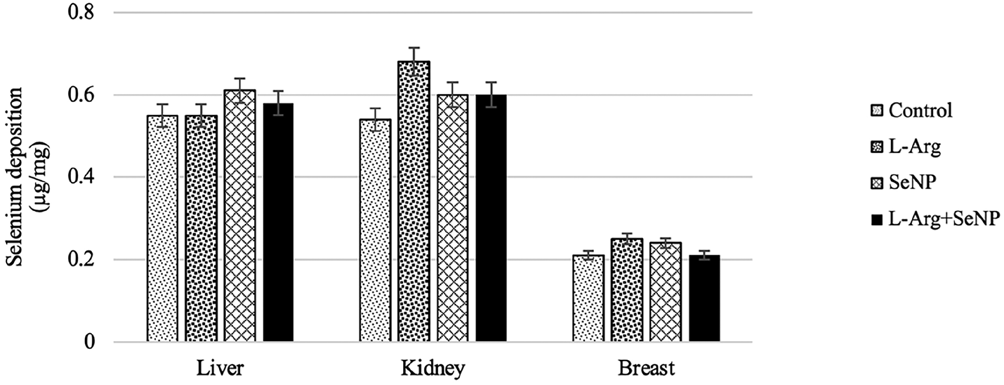
Effect of in ovo injection of l-arginine and selenium nanoparticles on selenium deposition. The data were presented as mean ± SEM (*n* = 16/treatment; 4 samples/replicate).

The differences in Se content among all treatment groups in the liver, kidneys, and breasts were not affected by treatment group (*P* > 0.05; Figure 3).

### GSH-Px activity in the breast muscle

**Figure 4.**
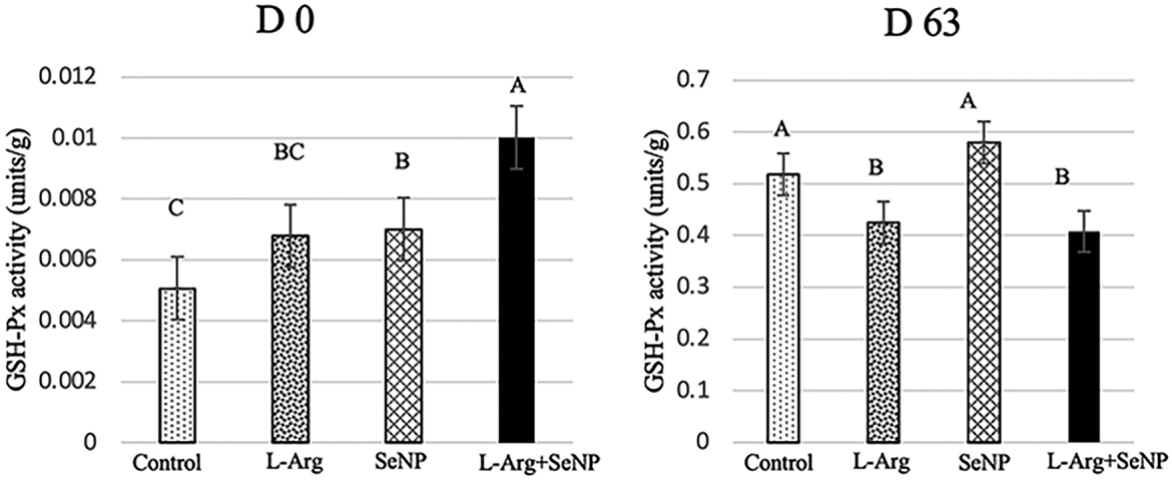
Effect of in ovo of l-arginine and selenium nanoparticles on GSH-Px activity in Korat chicken at days 0 and 63. A, B Means within column carrying no common superscripts are significantly different at *P* < 0.01. The data were presented as mean ± SEM (*n* = 16/treatment; 4 samples/replicate).

A significant difference in the GSH-Px activity was observed between the treatments (Figure 4). Compared to the control group, the highest GSH-Px activity was observed in the breast muscle of chickens treated with IOF_L-Arg + SeNP on the day of hatching (*P* < 0.01). On day 63, the GSH-Px activity levels in the breast muscle were significantly lower in the IOF_L-Arg and IOF_L-Arg + SeNP groups (*P* < 0.01) than in the control group.

## Discussion

KRC, a slow-growing chicken breed, is an alternative meat-type chicken breed in Thailand. Although it is recognized for its rich flavor and distinct texture, its relatively low growth rate and breast meat yield challenge its profitability. One promising solution to ensure highly efficient productivity is IOF, which targets embryonic myogenesis. Therefore, this present study aimed to evaluate the effect of in ovo feeding of L-Arg, SeNP, or a combination of L-Arg and SeNP on hatchability, post-hatching performance, muscle growth and characteristics, antioxidant capacity, and meat quality in KRC slow-growing chickens.

In general, hatchability is required for in ovo feeding. As expected, no unfavorable effects on hatchability or BW at hatching between the IOF and control groups were observed in this study. Lee et al. (2014) and Gao et al. (2017) reported similar results. However, other studies have demonstrated that in ovo arginine (Zhang et al., 2017) and selenium injection (Joshua et al., 2016) substantially enhanced hatchability and BW at hatching in poultry. Several factors, such as the IOF method, depth of IOF injection, and time and site of IOF influence the inconsistent response of in ovo nutrient feeding on hatchability and BW at hatching (Ebrahimi et al., 2017; Coskun et al., 2018). Different injected concentrations and volumes of L-Arg also have a major impact on toxicity during embryogenesis. Subramaniyan et al. (2019) reported that birds injected with a higher L-Arg concentration (2500 μg) exhibited reduced hatchability, survivor rate, and hatching weight than those with a lower L-Arg concentration (100 μg). Excessive arginine intake can interfere with the balance of other amino acids, affecting their absorption, metabolism, and availability during embryogenesis, thereby limiting their capacity to produce energy for hatching (Toghyani et al., 2019). Furthermore, Se nanoparticles and organic Se are less toxic and more effective in reducing poultry mortality than inorganic Se (sodium selenite and selenate) because of their high bioavailability, high catalytic efficiency, and strong adsorption ability (Wang and Xu, 2008). Therefore, the findings of the present study suggest that administering L-Arg (1%) and SeNP, either alone or in combination, through the IOF method is non-toxic and not detrimental to embryogenesis.

Arginine is an essential amino acid that is widely recognized for its involvement in the metabolism of various nutrients essential for growth and development (Khajali and Wideman, 2010). Toghyani et al. (2019) demonstrated that the IOF of L-Arg in the embryonic phase increased FI and BWG in broilers. Other studies have explained that the increase in BWG by IOF of L-Arg might be attributed to the increase in amino acid demand of chicks during embryogenesis, leading to the activation of higher protein synthesis and lower protein degradation (Al-Daraji et al., 2012). Gao et al. (2017) suggested that the IOF of L-Arg positively influences growth performance by promoting intestinal development and gastrointestinal hormone release. Moreover, Nano-Se supplementation has been effectively shown to improve weight gain by regulating ROS balance (Zhou and Wang, 2011). Unexpectedly, the results of the present study do not agree with those of previous studies, in which no significant effect on FI, BWG, or 63-d-BW was observed in the IOF treatment groups compared to the control group. These results indicated that in ovo feeding with either L-Arg or L-Arg + SeNP may not have beneficial effects on growth and development. Interestingly, feed efficiency improved in response to either IOF_SeNP alone or IOF_L-Arg + SeNP although no growth-promoting effect was detected, which is consistent with the findings of Ibrahim et al. (2020). FCR reduction in these 2 groups may be attributed to the role of SeNP, which acts as an antioxidant and protects cells from oxidative stress (Ibrahim et al., 2020). Nano-Se has been suggested to boost immune and antioxidant capabilities by stimulating GSH-Px enzyme activity, which, in turn, improves the resistance to oxidation, β-oxidation, and elimination of free radicals, thereby increasing the weight gain and feed efficiency of chickens (Jianhua et al., 2000). The improvement in feed efficiency, as indicated by reduced FCR in chickens that received a combination of L-Arg and SeNP indicated an additive effect of these 2 additives.

L-Arg and its metabolites, i.e., NO and polyamines, have been reported to play a dual role in myoblast proliferation and fusion (Buono et al., 2012), which positively contribute to an increased total breast weight and myofiber diameter in avian species (Fernandes et al., 2009; Yu et al., 2018). Our results support that these previous findings; the highest density of muscle fibers was observed in IOF_L-Arg + SeNP chickens after hatching and in IOF_L-Arg chickens when they were 42 d old. Additionally, myofibers were larger in chickens treated with IOF_L-Arg when they were 14 and 63 d old, as compared to the control group. The findings of the present study suggest that the additional SeNP injection, coupled with L-Arg, exerts nutritional benefits to enhance myofiber cell number through the hyperplasia process after hatching. Similarly, the positive regulation of muscle formation by dietary nano-Se in grass carp fed a high-fat diet was demonstrated by Liu et al. (2023). Nano-Se increased protein deposition and muscle fiber density by increasing myoblast proliferation and differentiation through the activation of the target of rapamycin (**TOR**) and myogenic determining factor pathways. Se deficiency has been reported to increase ROS concentrations, which inhibit TORC1 pathway-mediated protein synthesis, thereby restricting the hypertrophy of skeletal muscle fibers in fish (Wang et al., 2023).

Larger muscle fiber diameter and area and lower muscle fiber density can determine the larger BW of chickens (Koomkrong et al., 2015). However, the breast muscle weight did not exhibit differences between treatments in this study, although chickens in the L-Arg-injected group exhibited a higher fiber cell number and greater fiber diameter than those in the control group during the rearing period. This is in agreement with the findings of Li et al. (2016), who revealed that an increase in NO activity did not always stimulate follistatin expression, the main mediator of myoblast fusion although the effect of L-Arg on myogenesis was largely mediated by the NO pathway, thereby resulting in decreased muscle mass in hatched broilers. Besides the NO pathways, arginine could promote myogenic differentiation and myotube formation, accompanied by arginine-stimulated cytoplasmic Ca^2+^ concentration and 5ʹadenosylmonophosphate-activated protein kinase activation under in vivo and in vitro conditions (Gong et al., 2021). This suggests that the NO-dependent pathway is not the only pathway that affects muscle growth. Hence, the results of the current study emphasize that L-Arg certainly benefits muscle formation but may not have an adequate effect to make a difference in building muscle tissue. However, whether the exact mechanisms of L-Arg and SeNP, alone or together, regulate myogenic proliferation, differentiation, and determination in poultry is still unclear. Therefore, further research is required to confirm these findings.

The biochemical composition of breast meat demonstrated that the IOF treatment did not affect the fat, ash, and collagen contents. The highest protein content was observed in the breast meat of chickens treated with IOF_L-Arg + SeNP. Increasing extracellular L-Arg concentrations regulate protein synthesis and metabolism of energy substrates (Joshua et al., 2016). Wang et al. (2022) demonstrated that L-Arg enhances protein accumulation through the L-Arg/NO/mTOR/p70S6K pathway. Available evidence also showed that the mechanism underlying the effect of Se on muscle protein content involves the change in iodothyronine deiodinase activity induced by selenoproteins, consequently controlling muscle energy metabolism and protein assimilation (Marković et al., 2018). Hence, it is reasonable to suppose that SeNP might improve the deposition of proteins by boosting the uptake of amino acids into muscle cells through the control of thyroid hormones.

Considering breast meat quality attributes, IOF treatments caused no negative response to meat color (L*), pH value, or cooking loss, whereas the IOF treatments affected the percentages of drip loss and shear force. Drip loss and shear force are important indicators of the water-holding capacity and meat tenderness, respectively. Compared to the control group, chickens receiving IOF_L-Arg and IOF_L-Arg + SeNP had a similar effect on improved water-muscle protein interaction, as evidenced by decreased drip loss percentage, revealing the benefit of embryonic L-Arg injection. Ma et al. (2010) used L-Arg to treat fattening pigs and found that arginine considerably reduced drip loss. L-Arg is associated with an oxidative process that suppresses the degradation of myofibrillar proteins from oxidative stress and increases water reserves among myofibrils, which may contribute to drip loss reduction (Oksbjerg et al., 2019).

In this study, L-Arg caused a decrease in drip loss, which may have led to high tenderness. However, L-Arg enhanced chicken meat hardness (as evidenced by the higher shear force in chickens treated with IOF_L-Arg). Our results are inconsistent with those of Chen et al. (2023) who observed decreased shear force values in arginine-supplemented female pigs. This inconsistency could be due to the shear force being determined by several factors, such as the type of muscle fibers, composition and content of the connective tissue, and muscle water-holding capacity (Yu et al., 2018). Overall, IOF with 1% L-Arg can be assumed to partially improve meat quality by increasing meat tenderness and hardness.

Serum biochemical profiles are typically used to determine the general health status of humans and animals. Total protein, albumin, and globulin are the most important plasma proteins, and they represent good indicators of the metabolic capacity of hepatic function (Pan et al., 2009). The increase in serum levels of this substance reflects hepatocellular damage, with leakage of its contents into the bloodstream (Andretta et al., 2012). Additionally, hypoproteinemia causes a reduction in albumin levels accompanied by a relative increase in globulins, which reduces the A/G ratio (Santhosh et al., 2007). Here, no changes were observed in the total protein, albumin, globulin, and A/G ratio, in the IOF_L-Arg, SeNP, and combined groups, indicating that IOF treatments were not toxic to the liver. Additionally, the uric acid level, the main waste product of purine metabolism, is an important indicator of renal toxicity (Farsi et al., 2013). The uric acid levels were unaffected. Thus, it is reasonable to assume that embryonically injecting L-Arg and SeNP did not cause any damage to the liver or kidneys.

Selenium is an essential trace element that prevents oxidative stress. As a key component of GSH-Px, Se catalyzes the conversion of hydrogen peroxide and fatty acid hydroperoxides to water and fatty acid alcohols using reduced GSH to prevent cell membranes against oxidative damage (Huang et al., 2012). The liver, kidney, and muscle appear to be the target tissues for Se metabolism, excretion, and storage, respectively, when there is a high Se intake (Jiakui and Xiaolong, 2004). Our results showed that SeNP was not deposited in the liver, kidney, or breast muscle in the SeNP-injected group compared to the other groups. The lack of a substantial difference in SeNP deposition in the kidney, liver, and muscle between the treatment groups in the present study may have resulted from more rapid metabolism. The increased metabolic rate may result in more free radicals being generated, which, in turn, increases the need for substances with antioxidant properties, such as the selenoenzyme GSH-Px, to neutralize these free radicals and prevent oxidative damage. Supporting our hypothesis, GSH-Px activity in 1-d-old chicks was higher in the SeNP-injected group than in the other groups. This indicates that the enzyme activity was significantly improved by the additional SeNP injection into the embryos, which was also observed by other authors (Zhou and Wang, 2011; Marković et al., 2018). However, we found that the chickens that received IOF_L-Arg + SeNP exhibited lower GSH-Px activity than the control group and the chickens that received IOF_SeNP alone when they were 63 d old. The effect of this enzyme may not be sustained as the chickens matured (Marković et al., 2018) and would likely depend on the metabolic needs of chickens (Daun and Åkesson, 2004). A possible reason for this is the reactivity of this enzyme to background Se in the diet, which enables the induction of Se-dependent GSH-Px activity at a nominal level (Upton et al., 2009). Overall, embryonically injecting SeNP may improve antioxidant levels during the starter period. The absence of residue may indicate a lack of risk of Se intoxication in animals and consumers.

In conclusion, this is the first study highlighting the substantial effects of in ovo feeding of L-Arg and SeNP on slow-growing post-hatching performance, health status, and meat quality, which provide an early nutritional strategy for the poultry industry. The current study indicates that in ovo feeding of L-Arg combined with SeNP resulted in improved feed efficiency and enhanced antioxidant capacity at hatch without any adverse effects on chicken hatchability, health, or subsequent growth. Furthermore, meat from chickens that received IOF_L-Arg + SeNP exhibited a preferable texture with a higher protein content. However, several questions regarding the effect of L-Arg and SeNP on myogenesis remain unclear in the current work, providing directions for future research.

## Abbreviations

A: albumin
BSA: bovine serum albumin
BW: body weight
BWG: body weight gain
EDS: energy dispersive spectroscopy
FCR: feed conversion ratio
FI: feed intake
G: globulin
GSH: glutathione
GSH-Px: glutathione peroxidase
H_2_O_2_: hydrogen peroxide
H_2_SO_4_: sulfuric acid
HNO_3_: nitric acid
IOF: in ovo feeding
KRC: Korat chicken
L-Arg: l-arginine
LHK: Leung Hang Khao
NO: nitric oxide
Se: selenium
SEM: scanning electron microscopy
SeNP: selenium nanoparticles
TOR: the target of rapamycin
